# Unpacking the Influence of Visual Density on Pizza Packaging: Sensory Expectations and Purchase Intentions

**DOI:** 10.3390/foods13162567

**Published:** 2024-08-17

**Authors:** Cong Sun, Yuechun Ding, Xing Meng

**Affiliations:** 1 Pan Tianshou College of Architecture, Art and Design, Ningbo University, Ningbo 315211, China; suncong@nbu.edu.cn (C.S.); 2211370004@nbu.edu.cn (Y.D.); 2 School of Business, Ningbo University, Ningbo 315211, China

**Keywords:** visual density, sensory expectations, purchase intention, food packaging, consumption frequency, sensory marketing, consumer behavior

## Abstract

Visual density, defined as the number of identifiable elements per unit area within a visual design, significantly influences consumer perceptions. This study investigates the effects of varying visual densities in pizza packaging, encompassing both food-related and decorative elements, on consumers’ expectations regarding taste and texture, ultimately influencing their purchase decisions. We conducted a controlled experiment where participants were presented with pizza boxes of differing visual densities. Participants rated their expectations regarding the taste and texture of the pizza, as well as their purchase intentions. Additionally, we measured consumption frequency to evaluate its moderating influence on the observed effects. Results indicate that high-visual-density packaging significantly heightened expectations of taste and texture, independent of the element’s nature—whether food-related or decorative. Enhanced sensory expectations fully mediated the relationship between visual density and purchase intentions. Additionally, high consumption frequency amplified the effect of high visual density on sensory expectations and purchase intentions. These findings contribute to sensory marketing theory by highlighting the importance of visual density in packaging design and the role of consumption frequency. They provide practical implications for food packaging strategies aimed at enhancing consumer experience and satisfaction.

## 1. Introduction

Packaging is ubiquitous and has evolved significantly from its primary function of protecting products from environmental damage to becoming a pivotal marketing tool that influences consumer behavior [1,2]. According to Hine (1995), the role of packaging has undergone a profound transformation, with its history revealing a shift from mere functional use to a powerful medium for conveying brand image and attracting consumer attention. This evolution highlights how packaging not only ensures product integrity but also plays a crucial role in the dynamics of market communication and consumer decision making [1]. Hine’s insights underscore the strategic importance of packaging in segmenting markets and enhancing consumer purchasing desire. In the food sector, packaging serves not just to extend shelf life and maintain product quality, but also aids in market segmentation and heightens consumer purchasing desire [3]. High-quality food packaging design can make a product stand out in the market through unique visual appeal and aesthetic characteristics [4,5,6]. Moreover, it can convey specific attributes of the product, such as health or natural properties [7,8]. Importantly, food packaging can stimulate consumers’ sensory experience expectations [9]. In the food sector, consumer choices are influenced by multiple factors, with the sensory attributes of the food itself playing a key role in their decision-making process. Factors such as the visual appearance, odor, and texture of food directly determine consumer acceptance [10,11,12,13,14]. Although the sensory characteristics of food are crucial for consumer choice, in a market filled with numerous food options, it is impractical for consumers to try each product individually, especially in the case of packaged foods [15]. In this context, the significance of packaging is particularly evident. Studies have shown that well-designed food packaging can stimulate consumers’ associative thinking, establishing expectations of the product’s sensory experience before actual tasting, thereby influencing their sensory evaluation of the food [9,16].

According to Piqueras-Fiszman and Spence (2014), the formation of food expectations involves multiple processes within the brain: psychological processes interpret sensory data; physiological processes, such as neurochemical reactions, respond to these interpretations; and physical processes prepare the body for digestion [9]. This integrated response in the brain combines past experiences and current sensory inputs to form expectations about food [17]. The existing literature typically divides the sources of these expectations into two categories: “extrinsic” and “intrinsic” cues of the product. Extrinsic cues refer to sources of information associated with but not part of the product, such as advertising and packaging; intrinsic cues, on the other hand, refer to inherent characteristics of the product itself, such as taste and color. Food packaging, as a type of extrinsic cue, has a significant impact on the formation of consumer expectations. Due to limited cognitive resources, the brain tends to prioritize information that is more relevant to the current context, rather than irrelevant information [18]. This means that relevant information is more likely to capture attention and, thus, dominate the expectation formation process [19,20]. Furthermore, appropriate information can reduce the uncertainty of expectations, enhancing the acceptance of the food [21]. However, this process is influenced by individual consumer experiences. For familiar products, validating expectations does not necessarily increase consumer satisfaction but may instead lead to a sense of boredom. Moderately atypical information can break expectations to some extent, sparking curiosity and thereby enhancing the attractiveness of the product. Yet, if the sense of deviation is too strong, it may produce a negative effect [22,23].

The existing literature has extensively explored how food packaging influences consumers’ sensory expectations from various aspects such as packaging color, shape, visual texture, and imagery. Research indicates a correlation between specific colors and basic tastes; for instance, red/pink is commonly associated with sweetness, yellow/green with sourness, and white/blue with saltiness [24]. Regarding color brightness, bright colors are typically linked to sweetness, whereas darker shades are associated with sourness or bitterness [25]. Additionally, rounded, symmetrical shapes are perceived as sweet, while angular, asymmetrical shapes might signify sourness [26,27]. Compared to white, orange packaging is believed to make juice taste sweeter [28]. Packaging not only conveys specific taste-related information but also information about the intensity of flavors, where round, bright, and less saturated colors signal a mild taste, whereas triangular, dark, and highly saturated colors indicate a richer flavor [29]. Furthermore, food packaging impacts consumers’ expectations about the texture of food; for example, angular patterns might lead consumers to associate this with the texture of carbonated drinks [30]. Studies on food packaging imagery reveal that consumers often project the attributes of the imagery on the packaging onto the product itself. Such imagery typically includes the product, ingredients that confer aroma or flavor, and other relevant thematic images. For instance, consumers tend to estimate the caloric content of the main product based on the decorative elements on the packaging [31]. Consumers also infer the sweetness of soft cheese based on the depicted ancillary food elements like quince [32]. Moreover, when product packaging displays natural ingredients that contribute to the main flavor, such as fruits, consumers deduce that the product contains more natural ingredients [33]. Additionally, research has examined the role of other indirectly related imagery elements, such as sharp flame icons that evoke associations with spiciness [34].

Beyond characteristics such as color, shape, and texture, visual density is a crucial attribute in the design of product packaging. Visual density refers to the number of identifiable elements per unit area within a visual design [35,36]. Some studies suggest that higher visual density in packaging, featuring more product imagery, tends to increase the perceived quantity of the product and enhance willingness to pay [37]. Conversely, other studies indicate that fewer visual elements on packaging (low visual density) evoke associations with “higher product purity and fewer additives”, thereby increasing payment willingness [38]. From a design psychology perspective, these differences could be attributed to variations in the composition of visual images—the former enhances visual density by adding images related to the food inside the packaging, while the latter reduces visual density by minimizing decorative elements unrelated to the food. Additionally, while visual density on packaging influences consumer expectations of the product quantity or raw material components, its impact on sensory characteristics such as taste and texture has not received adequate attention.

Therefore, this study aims to investigate how visual density in packaging, exemplified by pizza box packaging, affects consumer expectations of sensory attributes (such as taste and texture) of food, and, consequently, how it influences their purchasing decisions. Pizza boxes were chosen as the subject of this study because pizza exhibits diverse characteristics, evident in its rich assortment of toppings and variety in textures. This diversity allows us to explore the impact of visual density on expectations for as many flavors and textures as possible, thereby broadening the applicability of our findings [39,40].

Previous research has provided valuable insights into pizza packaging. For example, Shen et al. (2015) explored the visual orientation of pizza packaging, noting that triangular packaging, particularly downward-facing triangles, might better capture consumer attention [41]. Barbosa (2021) focused on the role of localized design features in pizza packaging, such as how the placement of content images, logos, and flavor specification elements affects consumer attention levels [4]. Compared to previous studies, our research examines how visual density on pizza packaging impacts consumer sensory expectations. Additionally, considering the potential impact of the “correlation between packaging imagery and the food itself”, this study deliberately differentiates between “elements related to the food itself” and “decorative elements unrelated to the food”. This distinction allows us to explore how different types of packaging visual density affect consumer expectations. Furthermore, we investigate the moderating effect of consumer purchase frequency on the impact of visual density. Our study aims to deepen our understanding of the cross-sensory influence of food packaging and provide valuable insights for businesses regarding food packaging design.

## 2. Hypothesis Development

Consumers tend to use visual cues on packaging to gather information to assess the product’s value [42]. Mednick’s theory of remote associations suggests that creative thinking can connect seemingly unrelated elements to fulfill specific needs or objectives [43]. Moreover, certain studies have indicated that appropriate associative interventions can help reorganize elements targeted at utility, fostering creative associations [44]. For example, research has shown that consumers estimate the quantity of products inside the packaging based on the number of units displayed (e.g., the depiction of three versus fifteen pretzel crisps) [37]. This process exemplifies the psychological association journey from packaging cues to product attributes. Additionally, research has highlighted the role of information richness in reducing uncertainty and facilitating expectation formation [21]. Given that higher visual density in food packaging represents more visual information, we hypothesize that greater visual density can enhance expectation formation. The more decorative and visual elements there are included in product packaging, the higher the visual density, allowing consumers to associate and connect with more visual elements. These rich visual elements provide more inspiration and guidance, stimulating their associations and imaginations regarding the food’s characteristics. These associations, acting as mediators, help consumers build a mental model of the sensory properties of the food, such as taste and texture. Through these associative stimulations, consumers can develop a richer network of mental associations, leading to heightened expectations and preconceptions about the food, and thereby enhancing their anticipation of the food’s richness. Therefore, we propose that food packaging with high visual density can offer more visual stimulation and creative inspiration, helping consumers form more elaborate expectations about the possible sensory characteristics of the food, such as taste and texture.

Based on the above, we propose the following hypothesis:

**H1:**  *Compared to food packaging with low visual density, high-visual-density packaging can increase consumers’ expectations of the sensory characteristics of food, whether in terms of taste or texture.*

The diverse flavors and textures of food represent an increase in consumer choice and the diversification of food options. Consumers can choose from a variety of foods characterized by different tastes and textures, making it easier to find options that match their unique preferences [45]. To mitigate or prevent the tedium and diminishing hedonic returns of repeated consumption, consumers often actively seek novelty or diversity to maintain a sense of freshness [46]. Businesses cater to this desire for novelty by providing diverse choices, which can help slow the onset of consumer fatigue [47]. High visual density in packaging that evokes associations with various food flavors and textures serves as a means to create novel and interesting sensory experiences. This demand for novelty can stimulate consumers’ desire to try new foods, fulfilling their innate needs for stimulation and exploration [48,49]. Moreover, diversity as a compensatory control behavior reflects freedom of choice and autonomy, enhancing one’s sense of personal control [50]. Seeking diverse options is also a way to express individuality; choosing diverse foods helps to project an open and unique image [51]. Overall, for individual consumers, seeking diversity is a psychological manifestation of their pursuit of novelty, choice freedom, and utility maximization, which fosters a positive consumption willingness towards food.

Therefore, we propose the following hypothesis:

**H2:**  *The richness of sensory characteristics expected from food mediates the impact of visual density on consumer food purchase intention. Specifically, high-visual-density packaging is hypothesized to enhance consumers’ expectations of food’s sensory characteristics, thereby increasing their willingness to buy.*

Oliver and Winer (1987) emphasized the significant role of experience in expectation formation [52]. Research shows that the more familiar consumers are with a specific product, the more definite their expectations [53]. A higher frequency of consumption reflects more relevant experience and greater cognitive capability, which aids in expectation formation. Additionally, studies have indicated that experience is a foundation for generating novel ideas [54]. Consumers with a higher frequency of food consumption possess a higher level of domain expertise, thereby exhibiting more professionalism in such food categories. These consumers have a more systematic understanding of related concepts, forming tight associative networks and retaining more information in memory. This enables them to use a more in-depth, conceptualized structure for encoding and categorizing information, integrating different attributes and establishing connections, thus expanding the connotation of information [55,56]. Therefore, consumers with a higher frequency of consumption can more effectively make taste associations based on visual information, perform conceptual integration, and form comprehensive judgments about food. In contrast, consumers with lower frequency of consumption have more loosely organized knowledge, rely more on surface features for categorization, and depend on specific attribute knowledge to form judgments, making it difficult to infer more conclusions from limited packaging information.

Thus, we propose the following hypothesis:

**H3:**  *Consumption frequency moderates the impact of visual density in food packaging on expectations of food’s sensory characteristics. Specifically, consumers with higher food consumption frequency are more likely to associate high-visual-density packaging with a richer array of food sensory characteristics.*

**Figure 1:**
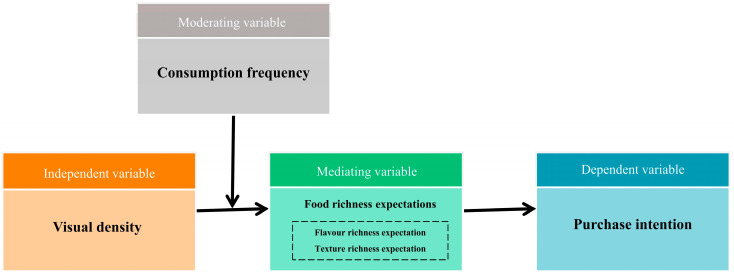
Hypothetical conceptual model.

Based on the analysis above, the theoretical framework of this study is summarized in Figure 1.

## 3. Research Design

### 3.1. Preliminary Experiments

Prior to the main study, we executed a series of preliminary tests to validate the suitability of our experimental materials and settings. To account for the potential impact of packaging images on food perception, we categorized images by their relevance to food, which facilitated manipulation of visual density in later tests. Initially, we evaluated how closely the decorative patterns on pizza boxes related to actual food items, ensuring diverse levels of food relevance across experimental materials. Subsequently, we measured the visual density of these patterns to ascertain significant variations. Lastly, we verified the realism and appropriateness of the experimental scenarios to enhance the external validity of our study.

#### 3.1.1. Image Relevance

**Figure 2:**
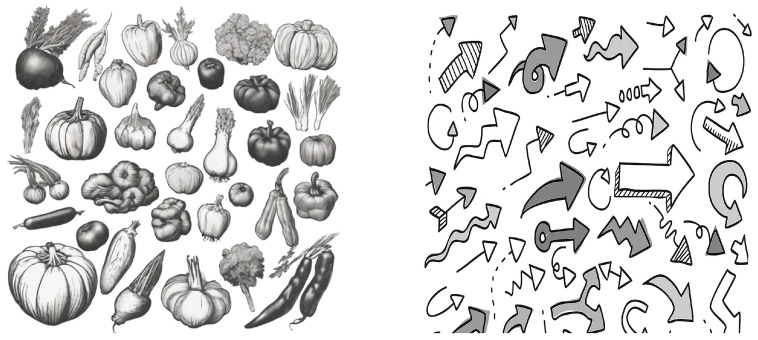
Packaging images.

To ascertain significant differences in the food-relevance of our experimental materials, we conducted this preliminary experiment. The high-visual-density packaging featured decorative patterns, as shown in Figure 2, with the left image being “food-related” and the right image “unrelated to food”. A total of 30 participants (11 males) were involved in this preliminary study. They were asked to rate the food relevance of the two sets of images. Specifically, participants responded to the question “How relevant do you think this image is to food?” on a scale from 1 (not relevant at all) to 7 (very relevant). Results of a one-sample t-test indicated that the scores for the left image were significantly higher than the neutral value of 4 (M = 6.83, SD = 0.144, t(29) = 40.941, *p* < 0.001). Conversely, the scores for the right image were significantly lower than the neutral value of 4 (M = 1.30, SD = 0.493, t(29) = −21.060, *p* < 0.001). This suggests that the manipulation of food relevance in Figure 2 was successful.

#### 3.1.2. Manipulation of Visual Density

**Figure 3:**
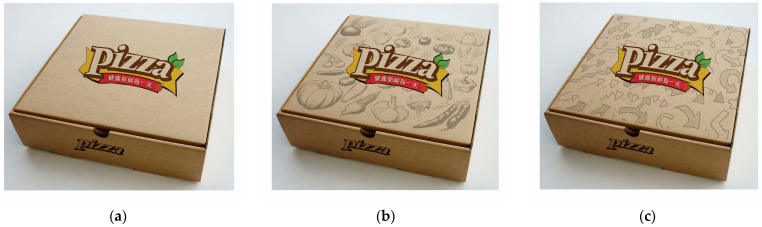
Stimulating materials. The Chinese advertising slogan “健康新鲜每一天” on the pizza box can be translated as “Fresh and Healthy Every Day”. (**a**) Low visual density; (**b**) high visual density with relevant elements; (**c**) high visual density with irrelevant elements.

Drawing on previous studies on visual density [57], we designed three sets of experimental stimuli, as depicted in Figure 3. To avoid the influence of brand effects on the experimental outcomes, we created a fictitious brand named “Pizza”. Since our research aimed to explore the impact of packaging visual density, the only differences among the three pizza box designs were in the density of decorative elements. All other aspects, including size, material, and packaging slogans, were kept consistent. A total of 162 participants (52 males) engaged in this preliminary experiment through an online platform. Participants were randomly assigned to view one of the images and were asked to rate “What do you think is the visual density of this packaging?” on a scale from 1 (low visual density) to 7 (high visual density). Analysis of variance revealed significant differences in perceived visual density among the three packaging conditions (M__low visual density_ = 2.490, M__high visual density with relevant elements_ = 4.231, M__high visual density with irrelevant elements_ = 3.814, F(2,159) = 21.29, *p* < 0.001). Visual density in Figure 3b,c was significantly higher than in Figure 3a, with no significant difference between Figure 3b,c.

#### 3.1.3. Scenario Setting

To enhance the participants’ attention and engagement, and to elicit natural responses through an immersive situational design, a heuristic scenario was set up. This scenario, drawn from everyday life, is described as follows: “You are contemplating what to have for lunch when, coincidentally, your roommate/colleague’s food delivery arrives. It’s a pizza, and you see its packaging”. To ensure this scenario setting reflects common real-life occurrences, a preliminary experiment was conducted to enhance the external validity of our study. Thirty participants (15 males) took part in this preliminary test. They were asked to evaluate how common the aforementioned scenario is in daily life by answering three questions: “I think it’s just a part of the daily routine”, “I think it’s something that happens every day in my life”, and “I think it’s a natural occurrence in everyday life”. Each question was rated on a scale from 1 (strongly disagree) to 7 (strongly agree). One-sample t-tests were conducted on the results for these three questions. The findings revealed that the average score for the first question significantly exceeded 4 (M = 5.27, SD = 1.375, t(29) = 5.917, *p* < 0.001), the second question scored significantly above 4 (M = 6.30, SD = 0.769, t(29) = 14.366, *p* < 0.001), and the third question also scored significantly above 4 (M = 5.97, SD = 1.068, t(29) = 10.424, *p* < 0.001). These results indicate that the scenario used in our experiment is representative.

### 3.2. Participants

A priori sample size estimation was conducted using G*Power 3.1 software, which indicated that a minimum total sample size of 160 was required to achieve 95% statistical power at a significance level of α = 0.05 and an effect size f of 0.15. Ultimately, 295 participants from China were recruited online for this experiment, including 63 males. The educational background of the participants was distributed as follows: 7 individuals (2.37%) with high school/vocational school, 18 (6.10%) with associate degrees, 141 (47.80%) with bachelor’s degrees, 120 (40.68%) with master’s degrees, and 9 (3.05%) with doctoral degrees.

This study strictly adhered to the Helsinki Declaration and the ethical guidelines of Ningbo University. It was noninvasive and did not involve the collection of any human or physiological data. Prior to the commencement of the experiment, all participants signed informed consent forms, where they were thoroughly informed about the purpose, process, and potential risks of the study, ensuring that they were fully aware of the content and participated voluntarily. Additionally, the collected data were anonymized to protect the privacy and security of the participants’ personal information.

### 3.3. Experimental Procedure

The formal experiment was conducted via Credamo, an online professional survey platform. Participants were randomly assigned to one of three different packaging condition groups. At the beginning of the experiment, participants filled out basic information, read and understood the instructions, and signed an informed consent form. Subsequently, participants were asked to imagine themselves in a scenario that had been tested and validated in the pre-experiment. Next, participants viewed the corresponding pizza packaging and were asked to answer questions regarding their expectations of the food’s richness. Specifically, participants responded to “I think the taste of this pizza is (1 = bland, 7 = rich)” and “I think the texture of this pizza is (1 = bland, 7 = rich)”. Additionally, participants were required to select from a list of options what flavors or textures they thought the pizza might possess. Specifically, they were asked to select “Seeing this pizza packaging, I associate it with possible flavors of tomato, sweet and spicy, seafood, savory, sweet, cheese (multiple choices allowed)” and “Seeing this pizza packaging, I think the pizza might feel soft, fine, smooth, crunchy, chewy, hard, grainy (multiple choices allowed)”. Then, participants were asked to rate the frequency of their pizza consumption (1 = never eaten, 2 = at least once a year, 3 = at least once a month, 4 = at least once a week, 5 = at least once a day). Participants also evaluated their willingness to purchase: “I would choose to buy food from this brand (1 = strongly disagree, 7 = strongly agree)”. Finally, appropriate compensation was provided to the participants for their time.

### 3.4. Data Analysis Methods

The data analysis for this study was divided into three main parts.

The first part aimed to explore the impact of visual density of food packaging on consumers’ taste and texture expectations. For this purpose, we employed a one-way analysis of variance (ANOVA) to test for significant differences in taste and texture expectations under different packaging conditions.

The second part aimed to investigate the role of anticipated richness of food as a mediating variable in the influence of visual density on purchase intention. Here, we utilized a three-step regression approach to test for the mediating effects of food richness expectations and employed the Bootstrap method for robustness checks to confirm the significance of the mediation effects.

The third part aimed to examine the moderating role of food consumption frequency. In this part, we tested the moderation effect by introducing interaction terms into a multiple regression model.

## 4. Research Results

### 4.1. Impact of Visual Density

#### 4.1.1. Influence of Visual Density on Sensory Expectations of Food

**Figure 4:**
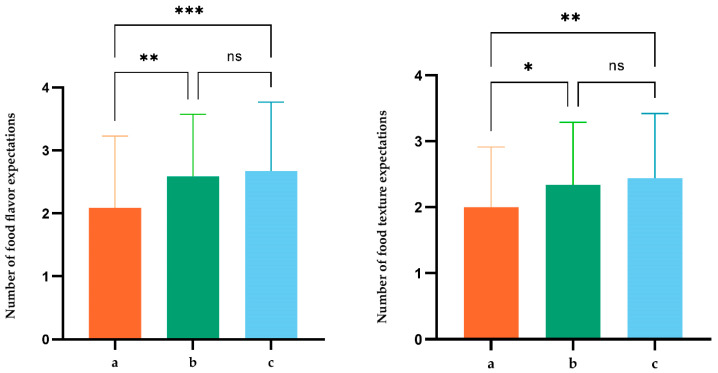
Expected flavor and texture counts in different packages. “a, b, and c” represent the low-visual-density group, the high visual density with relevant elements, and the high visual density with irrelevant elements, respectively. Statistical significance was assessed using ANOVA with Bonferroni correction to adjust for multiple comparisons, where *: *p* < 0.05; **: *p* < 0.01; ***: *p* < 0.001; ns: *p* > 0.05 indicates non significance.

We conducted an analysis of variance (ANOVA) using the three different packaging types from Figure 3 as independent variables, and the participants’ reported expectations for flavor and texture as dependent variables. The results are displayed in Figure 4, where the left graph presents the ANOVA results for “ flavor” as the dependent variable, and the right graph for the “texture” of the food.

For the flavor of pizza, there were significant differences in the expected number of flavor varieties among the three different packaging conditions (M__low visual density_ = 2.090, M__high visual density with relevant elements_ = 2.674, M__high visual density with irrelevant elements_ = 2.590; F(2,292) = 8.516, *p* < 0.001). Both the high-visual-density groups, whether with relevant or irrelevant elements, significantly outperformed the low-visual-density group in terms of expected flavor varieties (*p* < 0.001). However, there was no significant difference in the expected number of flavor varieties between the high-visual-density groups with and without relevant elements (*p* > 0.1).

Similarly, for the texture of pizza, there were significant differences in the expected number of texture types reported by participants under the three different packaging conditions (M__low visual density_ = 2.000, M__high visual density with relevant elements_ = 2.442, M__high visual density with irrelevant elements_ = 2.340; F(2,292) = 5.926, *p* < 0.01). Like the findings for taste, participants’ expectations for the number of pizza texture types were significantly higher in the high-visual-density groups, regardless of whether the packaging elements were food-related or not, compared to the low-visual-density group (*p* < 0.01). There was no significant difference between the high-visual-density groups (*p* > 0.1).

These results support Hypothesis 1, suggesting that, compared to low-visual-density food packaging, high-visual-density packaging can increase consumers’ sensory expectations, both in terms of the flavor and texture of the food.

#### 4.1.2. Influence of Visual Density on Purchase Intention

**Figure 5:**
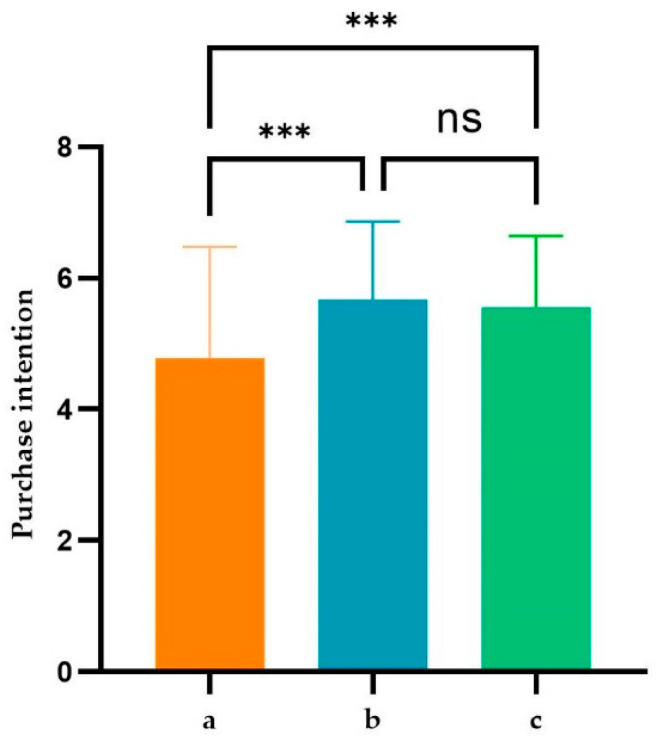
Purchase intention in different packages. “a, b, and c” represent the low-visual-density group, the high-visual-density group of relevant elements, and the high-visual-density group of irrelevant elements, respectively. Statistical significance was assessed using ANOVA with Bonferroni correction to adjust for multiple comparisons, where ***: *p* < 0.001; ns: *p* > 0.05 indicates non significance.

Using the same methodology as described in the previous section, we conducted another analysis of variance with participants’ reported purchase intentions as the dependent variable. The results are presented in Figure 5. Similar to the findings in Figure 4, there was a significant difference in purchase intentions for pizza among different participant groups (M__low visual density_ = 4.780, M__high visual density with relevant elements_ = 5.674, M__high visual density with irrelevant elements_ = 5.560; F(2,292) = 12.77, *p* < 0.001). Specifically, participants from the “high visual density with relevant elements” and “high visual density with irrelevant elements” groups reported significantly higher purchase intentions than those in the low-visual-density group (*p* < 0.001). However, within the high-visual-density groups, regardless of whether the packaging elements were relevant to the food, there was no significant difference in reported purchase intentions (*p* > 0.1).

### 4.2. Testing Mediating Effects

#### 4.2.1. Mediating Effect under the Condition with Relevant Elements

To verify the role of “expectations of sensory richness” as a mediating variable, we conducted a multivariate regression analysis using the three-step mediation model to test our hypotheses. First, visual density was converted into a dummy variable (0 = low-visual-density group, 1 = high-visual-density group with relevant elements). Then, using visual density as the independent variable, purchase intention as the dependent variable, and “expectations of sensory richness” as the mediator, we conducted the mediation effect test, controlling for the impact of gender and education level in the analysis. The results are shown in Table 1.

In Model 1, the visual density of food packaging significantly positively affected consumer purchase intentions (β = 0.842, *p* < 0.001). In Model 2, the visual density significantly positively impacted consumers’ “expectations of sensory richness” (β = 1.143, *p* < 0.001). In Model 3, when “expectations of sensory richness” were included as a mediator, the direct effect of visual density on purchase intentions was no longer significant, while “expectations of sensory richness” significantly positively predicted purchase intentions (β = 0.683, *p* < 0.001).

This indicates that “expectations of sensory richness” fully mediated the impact of visual density on purchase intentions, supporting Hypothesis H2. To enhance the robustness of the results, we also used a bias-corrected Bootstrap method for testing and found the direct effect to be nonsignificant with a coefficient of 0.062 and a 95% confidence interval ranging from [−0.247, 0.370], which includes zero. Concurrently, the mediating effect of “expectations of sensory richness” was significant, ab = 0.781 with a 95% confidence interval of [0.503, 1.059].

#### 4.2.2. Mediating Effect Under the Condition with Irrelevant Elements

We continued to verify the mediating role of expected richness of food sensory characteristics under conditions with irrelevant elements using the same method as the previous section. Initially, visual density was coded as a dummy variable (0 = low-visual-density group, 1 = high-visual-density group with irrelevant elements). We then used visual density as the independent variable, purchase intention as the dependent variable, and “expectations of sensory richness” as the mediator, while controlling for gender and education level in the analysis. The results are presented in Table 2.

Model 1 showed that visual density of the packaging significantly positively impacted consumer purchase intentions (β = 0.721, *p* < 0.001). Model 2 indicated that visual density significantly positively affected consumers’ “expectations of sensory richness” (β = 1.136, *p* < 0.001). In Model 3, after introducing “expectations of sensory richness” as a mediator, the direct impact of visual density on purchase intention was no longer significant, while “expectations of sensory richness” significantly positively predicted purchase intentions (β = 0.635, *p* < 0.001). This suggests that “expectations of sensory richness” fully mediated the effect of visual density on purchase intentions, supporting Hypothesis H2.

To enhance the robustness of these findings, we also employed a bias-corrected Bootstrap method for testing. The direct effect was found to be nonsignificant, with a coefficient of 0.000109 and a 95% confidence interval ranging from [−0.313, 0.313], including zero. Furthermore, the mediating effect of “expectations of sensory richness” was significant: ab = 0.721, with a 95% confidence interval of [0.476, 0.965].

### 4.3. Testing for Moderation Effects

**Figure 6:**
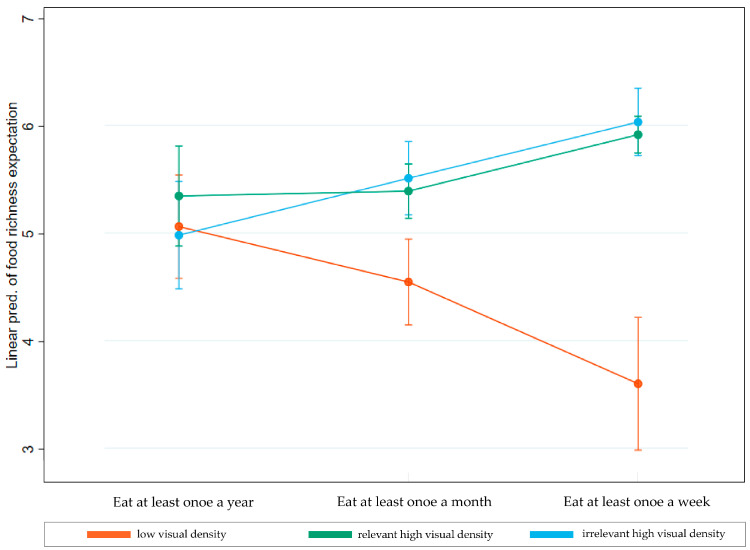
The visualization of the moderating effects. Error bars indicate the 95% confidence intervals for each mean value.

To examine the moderating effect of consumption frequency on the impact of visual density of food packaging, we converted the type of packaging into a dummy variable (0 = low-visual-density group, 1 = high-visual-density group with relevant elements, 2 = high-visual-density group with irrelevant elements), and food consumption frequency was also converted into a dummy variable (0 = at least once a year, 1 = at least once a month, 2 = at least once a week). Then, using expected richness of food sensory characteristics as the dependent variable, and food packaging, food consumption frequency, and their interaction terms as independent variables, we conducted a regression analysis. The results of the regression were visualized, as shown in Figure 6.

For participants who consume pizza at least annually, the expected sensory richness does not vary significantly across the three pizza packaging types. However, as the frequency of pizza consumption increases, significant differences gradually emerge between low- and high-visual-density packaging in terms of expected richness of food sensory characteristics, and these differences grow larger with increasing consumption frequency. Nevertheless, regardless of consumption frequency, within the high-visual-density packaging group, whether the decorative elements on the pizza packaging are food-related or not, there is no significant difference in participants’ expected richness of food sensory characteristics.

### 4.4. Summary of Experimental Results

This study explored the impact of visual density in food packaging on consumer expectations of sensory characteristics and purchasing intentions. The findings are as follows:

Firstly, high visual density in food packaging significantly enhanced consumer expectations regarding the richness of food flavors and textures. This effect was evident in packaging with both relevant and irrelevant decorative elements, although differences in evoking specific tastes and textures were observed.

Secondly, the study confirmed the role of “expectations of sensory richness” as a mediating variable between visual density and purchasing intentions. Specifically, high-visual-density packaging enhanced consumer expectations of sensory characteristics, which in turn increased purchasing intentions.

Lastly, the research revealed the moderating effect of food consumption frequency on the impact of visual density. Individuals with higher consumption frequencies exhibited more pronounced expectations of sensory characteristics from high-visual-density packaging. This effect was consistent across packaging with both relevant and irrelevant elements, but the trend intensified with increasing consumption frequency.

These findings not only enrich the theoretical framework of visual marketing but also provide practical guidance for food packaging design.

## 5. Conclusions and Discussion

### 5.1. General Conclusions

This study aimed to investigate how visual density in food packaging influences consumer sensory expectations and to further explore the impact of these expectations on purchasing intentions. Additionally, we examined the moderating effects of different types of packaging elements and consumer consumption frequency in this process. Through experimental research on pizza packaging, we derived the following main conclusions:

Firstly, the study confirmed a significant impact of visual density on sensory expectations. Specifically, high visual density in food packaging substantially enhanced consumers’ expectations about the taste and texture of the food. This effect occurred regardless of whether the packaging’s graphic elements were food-related, demonstrating that high visual density could evoke richer sensory expectations compared to low visual density. This finding not only supports Hypothesis 1 but also expands our understanding of the relationship between visual density and sensory expectations.

Secondly, this study found that consumers’ expectations of the richness of food sensory characteristics fully mediated the effect of visual density on purchase intentions. High-visual-density packaging enhanced consumers’ sensory expectations, which in turn significantly increased their willingness to purchase. This outcome supports Hypothesis 2 and illustrates the critical role of sensory expectations in the influence of visual design on consumer decision making.

Thirdly, whether the graphic elements on the packaging were related to the food did not significantly impact consumers’ sensory expectations or purchase intentions. In other words, although high visual density does enhance sensory expectations and purchase intentions, the relevance of the graphic elements is not a decisive factor. This insight provides new perspectives on image selection in packaging design, suggesting that visual density is more crucial than image relevance in enhancing sensory expectations and purchase intentions.

Lastly, this study unveils the moderating role of consumers’ food consumption frequency on the effects of visual density. Specifically, consumers with higher food consumption frequencies exhibit heightened sensitivity to packaging with high visual density. This sensitivity is evident regardless of whether the visual elements are food-related or not; packaging with high visual density significantly enhances their sensory expectations and purchase intentions. In contrast, for consumers with lower consumption frequencies, the impact of different packaging conditions on their sensory expectations and purchase intentions shows minimal variance. These findings support Hypothesis 3 and underscore the crucial moderating role of consumption frequency in the relationship between visual density and sensory expectations, as well as purchase intentions.

### 5.2. Theoretical Contributions

This study provides targeted insights into the specific role of visual density in food packaging, contributing nuanced additions to existing sensory marketing theories. Our findings indicate that increased visual density can significantly enhance consumer expectations regarding the sensory attributes of food, such as taste and texture. This effect persists regardless of whether the visual elements are directly related to the food content, challenging the conventional emphasis on content relevance in sensory marketing.

The specific contributions of this study include:

(1) Refining the scope of visual density: Unlike broad treatments of visual elements in prior research, this study uniquely isolates visual density as a distinct factor influencing consumer perceptions. Our results suggest that visual density alone, independent of content relevance, can alter consumer expectations and decision-making processes. This contributes to a more detailed understanding of the individual components of visual design in packaging.

(2) Challenging existing assumptions: The findings challenge existing marketing strategies that heavily rely on the relevance of visual elements to the product. The implication that nonrelevant, high-density visual elements can also significantly impact consumer expectations suggests a reevaluation of packaging design strategies that prioritize relevance over visual appeal.

### 5.3. Practical Implications

The practical implications of this study offer actionable insights for the food packaging industry, particularly in enhancing the sensory appeal of packaging to boost consumer interest and purchase behavior.

(1) Design recommendations: Designers are encouraged to experiment with high visual density in packaging, even when the elements are not directly related to the product. This study shows that such designs can still effectively enhance consumer expectations of taste and texture, potentially leading to increased purchase intentions.

(2) Enhanced consumer experience: Brands might use visually dense packaging as a strategy to enhance consumer engagement and satisfaction, particularly in unboxing experiences, which are crucial in e-commerce settings. This approach could differentiate products in a crowded market.

### 5.4. Limitations and Future Research Directions

While this study has made significant findings regarding the impact of visual density in food packaging on consumers’ sensory expectations and purchase intentions, there are several limitations that suggest potential directions for future research.

Firstly, the experimental design of this study employed grayscale patterns to manipulate visual density, aiming to mitigate the influence of color. However, the effect of color on consumer food perception has been well documented in extensive research [24,25,29]. Future studies could consider conducting experiments under different color schemes to explore the interaction between color and visual density on sensory expectations and purchase intentions. Moreover, this study exclusively used cardboard packaging, overlooking the effects of packaging material and glossiness. Previous research has indicated that different packaging materials, such as paper versus plastic, can generate varying expectations of product quality, taste, and healthiness [58,59]. Future research might further explore the impacts of visual density under different material and glossiness conditions on consumer behavior.

Secondly, this research focused on pizza as the experimental subject, a food item with rich attributes. While this choice aids in better understanding the relationship between visual density and attributes like texture and taste, the impact might be diminished for foods with more homogeneous tastes and textures. Therefore, future studies could consider selecting different types of food to verify the universality of the visual density effect.

Furthermore, the participants in this study were from China, and significant differences exist in dietary cultures and taste preferences across different countries and regions. Despite globalization increasing dietary diversity, the deep-rooted influence of local cultures on taste remains significant. Future research could be conducted in countries and regions with diverse cultural backgrounds to investigate the impact of visual density in food packaging on consumers’ sensory expectations and purchase intentions, thereby enhancing the external validity of the research findings.

In summary, this study has made significant progress in elucidating the mechanisms through which visual density in food packaging influences consumers’ sensory expectations and purchase intentions. However, it presents certain limitations. Future research could delve deeper into the diversity of colors and materials, the broadness of food types, and the universality across cultures to further enrich and refine the theoretical framework and practical guidelines in this field.

## Figures and Tables

**Table 1:** Hierarchical regression analysis to validate mediation effects under relevant elements condition.

Variable	Model 1	Model 2	Model 3
	Purchase Intention	Food Richness Expectations	Purchase Intention
Visual density	0.842 ***	1.143 ***	0.0618
	(0.197)	(0.199)	(0.157)
Food richness expectations			0.683 ***
			(0.070)
Intercept	6.199 ***	5.545 ***	2.411 ***
	(0.420)	(0.421)	(0.485)
N	195	195	195
F	13.09	18.32	34.42

Note: (1) Robust standard errors are in parentheses. (2) ***: *p* < 0.001.

**Table 2:** Hierarchical regression analysis to validate mediation effects under irrelevant elements condition.

Variable	Model 1	Model 2	Model 3
	Purchase Intention	Food Richness Expectations	Purchase Intention
Visual density	0.721 ***	1.136 ***	0.000109
	(0.187)	(0.180)	(0.159)
Food richness expectations			0.635 ***
			(0.077)
Intercept	6.451 ***	5.190 ***	3.158 ***
	(0.443)	(0.432)	(0.543)
N	200	200	200
F	14.24	18.79	28.86

Note: (1) Robust standard errors are in parentheses. (2) ***: *p* < 0.001.

## Data Availability

The original contributions presented in the study are included in the article, further inquiries can be directed to the corresponding author.
